# Antioxidant and *in vitro* anticancer activities of phenolics isolated from sugar beet molasses

**DOI:** 10.1186/s12906-015-0847-5

**Published:** 2015-09-07

**Authors:** Mingshun Chen, Hecheng Meng, Yi Zhao, Fuquan Chen, Shujuan Yu

**Affiliations:** College of Light Industry and Food Sciences, South China University of Technology, 381 Wushan road, Guangzhou, 510640 China; Guangdong Province Key Laboratory for Green Processing of Natural Products and Product Safety, South China University of Technology, Guangzhou, 510640 China

**Keywords:** Sugar beet molasses, Phenolic compounds, Antioxidant activities, Antitumor activities

## Abstract

**Background:**

In the present study, the phenolic compounds were prepared using ultrasonic-aid extraction from sugar beet molasses (SBM).

**Methods:**

Gallic acid (GA), cyanidin-3-O-glucoside chloride (CGC) and epicatechin (EP) were produced after column chromatography from the extraction, and further detected using NMR, QTOF-MS and ESI-MS/MS.

**Results:**

The three compounds exhibited strong antioxidant activities including DPPH radical scavenging activities, ABTS radical scavenging activities and ORAC values. GA showed the strongest antioxidant activity. Antitumor activities significantly increased in a dose-dependent manner. In particular, the CGC had growth inhibitory activities of 94.86, 87.27 and 67.13 % against the human colon (CACO-2), hepatocellular (HepG2) and breast (MCF-7) carcinoma cell lines, respectively, at the highest concentration of 400 μg/mL of the extracts. These results suggest that the three compounds are key chemical compositions valuable for preparing functional foods in the food industry.

**Conclusions:**

The results suggested that SBM is a natural source of antioxidant and antitumor agents for preparing functional foods.

## Background

Sugar beet is an important sugar crop cultivated for sugar production [1, 2]. Sugar beet molasses (SBM) is a by-product in sugar beet processing [3]. Early studies found that SBM can be used to produce alcohol and fermentation medium [4, 5]. While recent study reported that SBM showed higher antioxidant activity, anti-inflammatory and anti-proliferative activities [6, 7], the bioactive components of SBM are mainly phenolics, alkaloids, tannins, saponins, terpenoids, steroids, and volatile oil [8], and specific bioactive compounds such as syringic acid, vanillin, ferulic acid, hydroxybenzaldehyde, hydroxybenzoic acid, luteolin/kaempferol, feruloyl-arabinose-arabinose and caffeoyltartaric acid which have been prepared and identified [9].

Phenolic contents have been demonstrated to have a variety of bioactivities such as anti-aging, anti-fatigue, anti-hypoxia, immunological, anti-radiation, anti-inflammatory, anti-proliferative and hypoglycemic effects [10–14]. Meanwhile many investigations revealed that phenolic contents contribute to the antioxidant and antitumor activities of plants [15]. Antioxidants can restrict the deleterious effects of these oxidant reactions and these restrictions can involve scavenging free radicals or preventing radical formation [16]. However, to the best of our knowledge, there were few reports on the antioxidant and antitumor activities of phenolics from sugar beet molasses (SBMP). Given the rich natural resources of SBM, the extraction researches of SBMP will have a highly practical value.

The primary objective of the present study is to separate and purify the phenolic compounds in SBM, identify the structure of prepared phenolic compounds, and study the bioactivity of the prepared phenolic compounds in SBM.

## Methods

### Materials and reagents

SBM was provided by Xinjiang Green Xiang Sugar Industry Co., Ltd (Tacheng, China). 2,2-diphenyl-1-picryhydrazyl (DPPH), 2,2-azino-bis-(3-ethylbenzothiazoline-6-sulfonic acid) diammonium salt (ABTS), ascorbic acid (Vc), fluorescein, 2,2′-azobis (2-methylpropionamidine) dihydrochloride (AAPH), trolox [(±)-6-hydroxy-2,5,7,8-tetramethylchroman-2-carboxylic acid] and 3-(4,5-dimethylthiazol-2-yl)-2,5-diphenyltetrazolium bromide (MTT) were purchased from Sigma-Aldrich Chemical Co. (St. Louis, MO, USA). Dulbecco’s modified eagle medium (DMEM), penicillin-streptomycin were purchased from Gibco Co. (Long Shang Industry Park, Beijing, China). Fetal bovine serum (FBS) was purchased from Zhejiang Tianhang Biological Technology Co. (Zhejiang, China). Methanol (HPLC-grade) was purchased from Merck Co. (Whitehouse Station, NJ, USA). Ultra-pure water was prepared by a Milli-Q system (Millipore, Bedford, USA).

### Extraction, isolation and purification

The SBM (2.0 kg) was extracted by 30 mL 70 % (w/v) ethanol using a RK102H ultrasonic (BANDELIN SONOREX, Germany). Extraction conditions were ultrasonic power, 450 W, HCl concentrations 1.6 mol/L, temperature 40 °C and time 60 min. The extract was concentrated at 45 °C in vacuum using a rotary evaporator (RE-52 A, Yarong Co. Ltd., Shanghai, P. R. China) to obtain the total extraction fraction (634.02 g). Then, the total extracted fraction was suspended in distilled water. The resulting solution was successively partitioned with different solvents which yielded petroleum ether fraction, chloroform fraction, ethyl acetate fraction, *n*-butanol fraction and aqueous fraction. After drying *in vacuo*, five fractions were obtained. These fractions included the petroleum ether fraction (5.26 g, yield coefficient 0.83 %), chloroform fraction (32.97 g, yield coefficient 5.20 %), ethyl acetate fraction (76.7 g, yield coefficient 12.10 %), *n*-butanol fraction (58.71 g, yield coefficient 9.26 %), and water fraction (142 g, yield coefficient 22.40 %). In the biological activity screening tests, the ethyl acetate fraction showed stronger antioxidant and antitumor activities than other four fractions. Therefore, the ethyl acetate fraction was chosen for further purification.

**Fig. 1:**
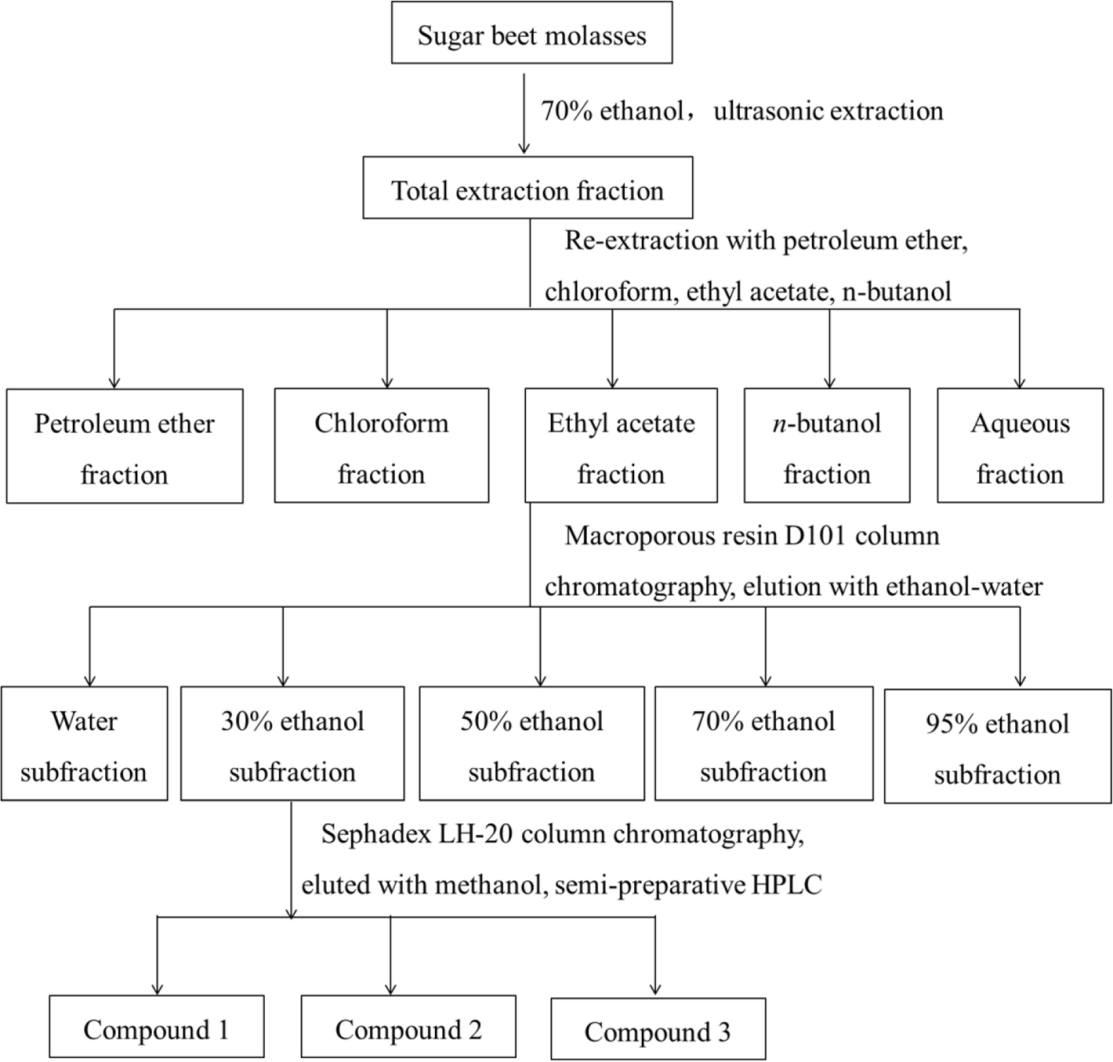
The extraction and separation procedure of *sugar beet molasses*

The ethyl acetate fraction (70.0 g) was loaded onto a column (3.5 cm × 100 cm) of macroporous resin D101, and the column was stepwise eluted with water, 30, 50, 70, and 95 % ethanol at a flow rate of 10 mL/min to yield five sub-fractions. After HPLC analyses, 30 % ethanol sub-fraction was further purified using a Sephadex LH-20 column chromatography and eluted with methanol, followed by a semi-preparative HPLC eluting with methanol/water, to yield **compound 1** (1293 mg), **compound 2** (241 mg) and **compound 3** (486 mg). The semi-preparative high performance liquid chromatography (HPLC) system consisted of a C_18_ column (RP_18,_ 10 μm, 250 mm × 20 mm), a Waters 600 pump and a Waters 2998 Diode Array Detector (DAD) (Waters, Milford, MA, USA) analyzed at 35 °C. The mobile phase was 1 % acetic acid aqueous solution (A) and methanol (B) with a gradient program of 0–10 min, linear gradient 5–10 % B; 10–70 min, linear gradient 10–20 % B; 70–90 min, 20 % B isocratic; 90–130 min, linear gradient 20–40 % B; 130–140 min, linear gradient 40–100 % B and 140–180 min 100 % B isocratic elution at a flow rate of 15 mL/min. T 2 mL of samples were injected. Absorption wavelengths were set at 280 and 360 nm. The extraction and separation procedure of SBMP is shown in Fig. 1.

### Identification of purity

Sample purity was analyzed using an HPLC-DAD system, which consisted of a C_18_ column (XBridge™ Shield, RP_18,_ 5 μm, 250 mm × 4.6 mm), a Waters 600 pump and a Waters 2998 DAD (Waters, Milford, USA), column temperature was set at 35 °C. The mobile phase was 1 % acetic acid aqueous solution (A) and methanol (B) with a gradient program of 0–5 min, linear gradient 5–10 % B; 5–35 min, linear gradient 10–20 % B; 35–45 min, 20 % B isocratic; 45–65 min, linear gradient 20–40 % B; 65–70 min, linear gradient 40–100 % B and 70–90 min 100 % B isocratic elution at a flow rate of 1 mL/min. The injection volume is 20 μL, and the detection wavelengths were set at 280 and 360 nm.

### Identification of compounds

The purified compounds from SBM were identified by electrospray ionization-mass spectrometry (ESI-MS), ^1^H NMR and ^13^C NMR spectrometry. The ESI-MS was carried out in a LCQ-Fleet mass spectrometer (Thermo Fisher Scientific, Waltham, MA), with an electrospray ionization source using a negative mode (*m/z* 50–800). ^1^H NMR spectra and ^13^C NMR spectra were recorded on a Bruker Avance DMX-500 spectrometer (Bruker Biospin GmbH, Germany), operating at 500 and 125 MHz for ^1^H and ^13^C, respectively, using D_2_O or deuterated dimethyl sulfoxide (DMSO-*d6*). In D_2_O, tetramethylsilane (TMS) was used as the internal standard. In DMSO-*d*_*6*_, the residual solvent was used as the internal standard. Chemical shifts were expressed in δ (ppm) downfield from TMS as an internal standard, and coupling constants were reported in hertz.

### Assay of total antioxidant activity

The scavenging activity on DPPH free radical (DPPH) was measured according to the reported method with some modifications [17]. 0.5 ml of sample solution was mixed with 2.0 ml DPPH methanol solution (6 × 10^−5^ mol/L) and the mixture was shaken vigorously and incubated in dark at 30 °C for 30 min. Then, the absorbance was measured at 517 nm. Deionized water and ascorbic acid (V_C_) were used as the blank and positive control, respectively. The ability to scavenge DPPH was calculated by the eq. 1:

1 $$ \mathrm{Scavenging}\ \mathrm{activity}\ \left(\%\right) = \left(1-\frac{Abs_1-{Abs}_2}{Abs_0}\right)\times 100 $$

where Abs_0_ is the absorbance of water instead of sample solution, Abs_1_ is the absorbance of the sample solution, and Abs_2_ is the absorbance of methanol instead of DPPH solution.

The ABTS radical scavenging activity was assessed according to the recent reports with some modification [18, 19]. Briefly, ABTS solution was produced by reacting 5 mL of ABTS (7 mM) and 5 mL of potassium persulphate (2.45 mM) for 12–16 h in the dark at room temperature. This mixture was then diluted with absolute ethanol to obtain an absorbance of 0.70 ± 0.02 at 734 nm before use. Then, 0.4 mL of tested sample at different concentrations was mixed with 3.0 mL of ABTS solution. The mixture was incubated for 6 min in he dark and the absorbance was measured at 734 nm against blank. Ascorbic acid (Vc) with the same concentration was used as a positive control. The ABTS scavenging activity was calculated using the eq. 2:

2 $$ \mathrm{Scavenging}\ \mathrm{activity}\ \left(\%\right)=\left(1-\frac{A_{\mathrm{s}}}{A_{\mathrm{c}}}\right)\times 100 $$

where *A*_c_ is the absorbance of control without sample, *A*_s_ is the absorbance of the sample.

The oxygen radical absorbance capacity (ORAC) assay was carried out according to the modified method of Ou et al. [20]. Briefly, sample solution was prepared with 75 mM phosphate buffer (pH 7.4). The 20 μL sample aliquots or Trolox standard (6.25–100 μM) were added in a black 96-well plates (Greiner Bio-one Cellstar, Frickenhausen), and then 200 μL of 95.6 nM fluorescein solution was added to each well. The mixture was incubated for 30 min at 37 °C. Finally, 20 μL of AAPH was automatically injected, and the microplate was shaken for 20 s before each measurement. The fluorescence was measured using a Varioskan Flash Multimode Reader (Thermo Fisher Scientific Inc.) at excitation of 540 nm and emission of 565 nm for 35 cycles every 3.0 min. The buffer was used as blank. The ORAC value (μmol Trolox equivalents (TE)/g) refers to the required Trolox content (μmol) when ORAC of Trolox is equal to 1 g sample.

### Determination of antitumor activity

Human colon (CACO-2), hepatocellular (HepG2) and breast (MCF-7) carcinoma cell lines were provided by the Medical College of Sun Yat-Sen University (Guangzhou, China). The cells were cultured in DMEM containing 10 % FBS, 100 μg/mL streptomycin and 100 U/mL penicillin in a humidified incubator with 5 % CO_2_ at 37 °C.

The antitumor activities of samples were evaluated using the MTT assay with some modifications [21]. Briefly, CACO-2, HepG2 and MCF-7 cells were harvested during the logarithmic growth phase, seeded in a 96-well plate (5 × 10^4^ cells/mL), and incubated at 37 °C in an atmosphere of 5 % CO_2_. After anchoring to the wells, additional medium (100 μL) containing different concentrations of test samples were added to each well. The cells were then incubated at 37 °C for 48 h in an atmosphere containing 5 % CO_2_. Then, 20 μL of MTT solution (5 mg/mL) was added to each well, and the incubation was continued for an additional 4 h. Then the supernatant was removed and 150 μL of DMSO was added to each well. The plate was shaken for 10 min to dissolve formazan crystals. The absorbance of the above DMSO solution was measured at 570 nm by a microplate reader. The buffer and 5-fluorouracil were used as the blank and positive control respectively. The inhibitory rate was calculated using the eq. 3:

3 $$ A=\left(1-\frac{A_{\mathrm{s}}-{A}_b}{A_{\mathrm{c}}-{A}_b}\right)\times 100\% $$

where *A* is the cancer cell growth inhibitory rate; *A*_c_ is the absorbance of the control; *A*_s_ is the absorbance of sample; and *A*_b_ is the absorbance of the blank.

### Statistical analysis

Data were analyzed using SPSS (SPSS Inc., Chicago, IL, USA) and presented as mean ± SD with triplicates. Significance was determined at *p <* 0.05 by analysis of variance (ANOVA) followed by Duncan’s least significant test.

## Results and discussion

### Structural identification

Three compounds isolated and obtained from SBM were identified as gallic acid (GA), cyanidin-3-O-glucoside chloride (CGC) and epicatechin (EP). Their spectroscopic data were listed below.

Gallic acid (Compound 1): C_7_H_6_O_5,_ white powder, purity, 99.2 %. QTOF-MS m/z: 177.0158 [M + Na-H_2_O]^+^, ESI-MS/MS m/z: 177, 153, 127. ^1^H-NMR (500 MHz, CDCl_3_) δ H: 7.12 (2H, s, H-2, 6). ^13^C-NMR (125 MHz, CDCl_3_) δ C: 121.2 (C-1), 109.4 (C-2), 145.3 (C-3), 138.6 (C-4), 145.3 (C-5), 109.4 (C-6), 168.5 (COOH) [22].

Cyanidin-3-O-glucoside chloride (Compound 2): C_21_H_21_O_11,_ dark brown crystalline powder, purity, 98.7 %. QTOF-MS m/z:450.1157 [M + H]^+^, ESI-MS/MS m/z:450, 287. ^1^H-NMR (CD3COCD3, 500 MHz) δ H: 8.92 (1H, s, H-4), 6.88 (1H, d, *J* = 2.0 Hz, H-6), 6.91 (1H, s, H-8), 8.10 (1H, d, *J* = 1.0 Hz, H-2′), 7.02 (1H, d, *J* = 8.5Hz, H-5′), 8.26 (1H, d, *J* = 7.5Hz, H-6′), 5.28 (1H, d, *J* = 4.5Hz, H-1″), 3.13 (1H, t, H-4″), 3.38 (1H, q, H-3″), 3.31 (1H, q, H-2″), 3.48 (1H, m, H-5″), 3.82 (1H, m, H-6a″), 3.71 (1H, m, H-6b″) [23].

Epicatechin (Compound 3): C_15_H_14_O_6,_ white crystalline powder, purity, 99.5 %. QTOF-MS m/z:291.0863 [M + H]^+^, ESI-MS/MS m/z: 291, 273, 165, 139. ^1^H-NMR (CD3COCD3, 400 MHz) δ H: 7.03 (1H, d, *J* = 2.0 Hz, H-2′), 6.82 (1H, d, *J* = 8.0 Hz, H-5′), 6.77 (1H, dd, *J* = 8.0, 2.0 Hz, H-6′), 5.99 (1H, d, *J* = 2.0 Hz, H-8), 5.89 (1H, d, *J* = 2.0 Hz, H-6), 4.86 (1H, s, H-2), 4.11 (1H, m, H-3), 2.85 (1H, dd, *J* = 16.4, 4.4 Hz, H-4), 2.72 (1H, dd, *J* = 16.4, 3.2 Hz, H-4). ^13^C-NMR(CD3COCD3, 100 MHZ,) δ C: 28.83 (C-4), 68.33 (C-3), 82.70 (C-2), 95.41 (C-8), 96.13 (C-6), 100.62 (C-10), 115.23 (C-5′), 115.70 (C-2′), 120.06 (C-6′), 132.14 (C-1′), 145.64 (C-3′), 145.72 (C-4′), 156.89 (C-9), 157.22 (C-5), 157.73 (C-7) [24, 25].

### Antioxidant activity

**Fig. 2:**
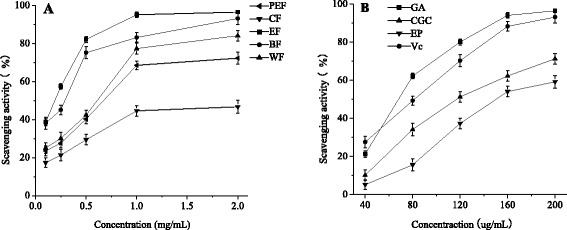
DPPH radical scavenging activities of each fraction and compound isolated from SBM. **a** PEF: petroleum ether fraction; CF: chloroform fraction; EF: ethyl acetate fraction; BF: *n*-butanol fraction; WF: water fraction; **b** GA: gallic acid, CGC: cyanidin-3-O-glucoside chloride; EP: epicatechin All results are the means ± SD (*n* = 3)

The DPPH radical scavenging activities of extractions, GA, CGC and EP were shown in Fig. 2. All fractions showed remarkable scavenging activity against DPPH radicals in a dose-dependent manner. The scavenging activity of the ethyl acetate fraction was higher than those of other fractions (Fig. 2a). GA showed higher scavenging activities against DPPH radicals than Vc. CGC and EP showed relatively lower scavenging activities (Fig. 2b).

**Fig. 3:**
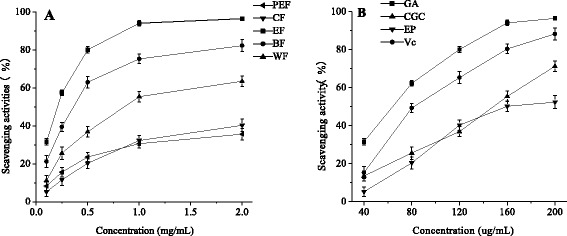
ABTS radical scavenging activities of each fraction and compound isolated from SBM. **a** PEF: petroleum ether fraction; CF: chloroform fraction; EF: ethyl acetate fraction; BF: *n*-butanol fraction; WF: water fraction; **b** GA: gallic acid, CGC: cyanidin-3-O-glucoside chloride; EP: epicatechin. All results are the means ± SD (*n* = 3)

The ABTS radical scavenging activities of each fraction, GA, CGC and EP were shown in Fig. 3. All fractions showed remarkable scavenging activity against ABTS radicals in a concentration-dependent manner. The scavenging activity of the ethyl acetate fraction was higher than those of other fractions (Fig. 3a). GA showed higher scavenging activities against ABTS radicals than Vc. CGC and EP showed relatively lower scavenging activities (Fig. 3b).

**Fig. 4:**
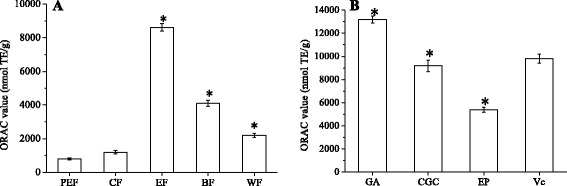
The ORAC values of each fraction and compound isolated from SBM. **a** PEF: petroleum ether fraction; CF: chloroform fraction; EF: ethyl acetate fraction; BF: *n*-butanol fraction; WF: water fraction; **b** GA: gallic acid, CGC: cyanidin-3-O-glucoside chloride; EP: epicatechin. All results are the means ± SD (*n* = 3). *p < 0.05, statistically significant in comparison with control

The oxygen radical absorbance capacities of samples were shown in Fig. 4. As shown in Fig. 4a, all the extraction fractions showed strong oxygen radical absorbance capacity. The ORAC values were in the decreasing order of ethyl acetate fraction > *n*-butanol fraction > water fraction > chloroform fraction > petroleum ether fraction. The ORAC values of three compounds are shown in Fig. 4b. GA showed stronger oxygen radical absorbance capacity than Vc while those of CGC and EP were relatively low.

According to the results of DPPH, ABTS radical scavenging and ORAC assays, the ethyl acetate fraction showed the strongest antioxidant activity among five extraction fractions. The three compounds isolated from the ethyl acetate fraction could be explored as natural antioxidants as they showed strong antioxidant activities. GA showed the strongest antioxidant activity, followed by Vc which is greater than those of the CGC while EP showed the least antioxidant activity.

The three compounds, GA, CGC and EP, obtained in our study are three typical phenolic compounds in plants which have been reported to possess strong antioxidant activity [26, 27]. According to reports, free hydroxyl groups in phenolics are mainly responsible for antioxidant activity [28, 29]. GA, CGC and EP, belonging to the phenolic compounds, are rich in multiple phenolic hydroxyl groups, which have been considered to be important antioxidants for a long time, and their antioxidant activities could be attributed to the numerous hydroxyl groups present on their structures. However, according to the literature [29, 30], the number of hydroxyl groups bonded to the aromatic ring and their positions are probably the most important but not the only factors influencing the antioxidant activities of phenolic components. Meanwhile, character of substituents (carboxyl or acetyl group) and their position in relation to the hydroxyl groups seem to influence the antioxidant or anti-radical features.

### Antitumor activity

**Fig. 5:**
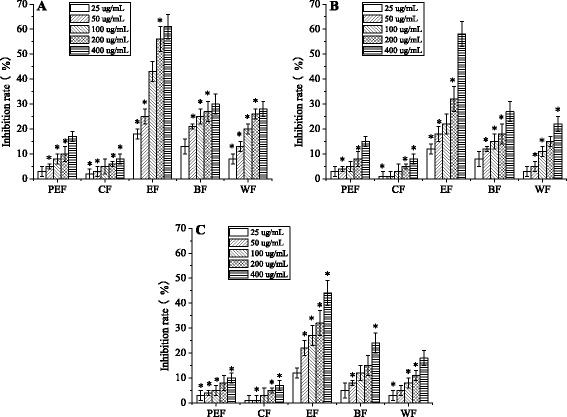
Inhibitory effects *in vitro* of five fractions against **a** CACO-2 cells, **b** HepG2 cells and **c** MCF-7 cells. PEF: petroleum ether fraction; CF: chloroform fraction; EF: ethyl acetate fraction; BF: *n*-butanol fraction; WF: water fraction. All results are the means ± SD (*n* = 3). *p < 0.05, statistically significant in comparison with control

**Fig. 6:**
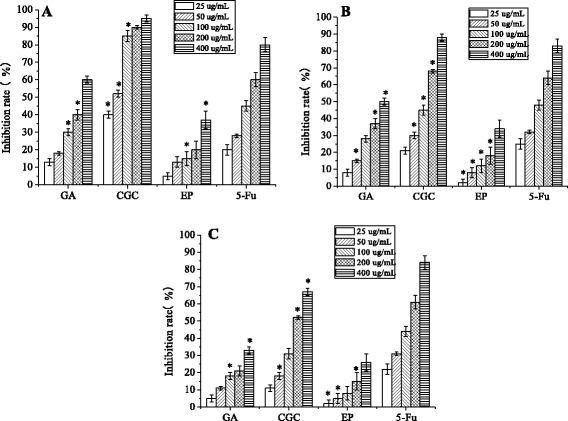
Inhibitory effects *in vitro* of three compounds and 5-fluorouracil (5-Fu) control against **a** CACO-2 cells, **b** HepG2 cells and **c** MCF-7 cells. GA: gallic acid, CGC: cyanidin-3-O-glucoside chloride, EP: epicatechin. All results are the means ± SD (*n* = 3). *p < 0.05, statistically significant in comparison with control

The antitumor activities of five fractions against cancer cells are shown in Fig. 5. Among the five extraction fractions, the ethyl acetate fraction showed the highest inhibitory effect on CACO-2 (Fig. 5a), HepG2 (Fig. 5b) and MCF-7 (Fig. 5c) cell proliferation at the concentrations ranging from 25 to 400 μg/mL. The inhibitory effect was displayed in a dose-dependent manner. As shown in Fig. 6, the inhibitory rates of the ethyl acetate fraction on CACO-2, HepG2 and MCF-7 cells were higher than other fractions. The degree of antitumor activities of the ethyl acetate fractions showed that active compounds may be more concentrated in the ethyl acetate fraction than may be present in other fractions.

As shown in Fig. 6, GA, CGC and EP showed significant antitumor activities. CGC exhibited the strongest inhibitory effect on CACO-2 (Fig. 6a), HepG2 (Fig. 6b) and MCF-7 (Fig. 6c) cells. The inhibitory rate of CGC (400 μg/mL) was about 95 %. Additionally, the inhibitory rates of CGC (all concentrations) were much higher than that of 5-fluorouracil. However, GA and EP showed relatively weak inhibitory effects on the CACO-2, HepG2 and MCF-7 cells, and the inhibitory rates increased slowly with the increase in their concentration. From previous studies, compounds with higher antioxidant activities always had higher antitumor activity and prevented the cellular senescence and apoptosis [9, 31]. However, although CGC showed lower antioxidant activity than GA, it had the strongest inhibitory effects on the cancer cell proliferation.

Early studies showed that GA, CGC and EP had anticancer activity. GA has been shown to have antitumor activity in many cancer cells without damaging normal cells [32–36]. Wang et al. [32] reported GA induced apoptosis by triggering the extrinsic or Fas/FasL pathway as well as the intrinsic or mitochondrial pathway in MCF-7 cells. Filipiak et al. [33] reported CGC showed inhibitory activity against gelatinases corresponding to its cytotoxic activity in HT1080 cells. Xu et al. [34] reported CGC attenuated ethanol-induced migration/invasion of breast cancer cells expressing high levels of ErbB2 (BT474, MDA-MB231 and MCF7ErbB2) in a concentration dependent manner. Zhao et al. [35] reported GA significantly decreased human cervical cancer cell proliferation and tube formation in human umbilical vein endothelial cells. Siddique et al. [36] reported EP could cause a decrease in the proliferation, guanosine triphosphate-bound Ras protein, Akt phosphorylation and NF-κB transcriptional activity of premalignant and malignant Krasactivated PDE cells. Though the antitumor activities of these compounds had been previously reported, the mechanism(s) of antitumor activity of the individual compound needs to be investigated. However, the presence of the three compounds may be responsible for the antioxidant and antitumor activities exhibited by SBM and suggested that SBM might be used as an additive in antitumor food.

## Conclusions

Three compounds, Gallic acid (GA), cyanidin-3-O-glucoside chloride (CGC) and epicatechin (EP) and the main compounds in SBM with high antioxidant and antitumor activities. GA possessed the strongest antioxidant activity. CGC showed strong antitumor activities against human colon (CACO-2), hepatocellular (HepG2) and breast carcinoma cells. The antitumor activity was higher than positive control (5-fluorouracil). Further studies will be needed to investigate other chemical compounds and bioactivity of SBM. These results suggest that SBM is a potential source of antioxidant and antitumor agents for preparing functional foods.
